# When Feeding Difficulties Are due to Genetics: The Case of Familial Partial 9q Duplication

**DOI:** 10.1177/2324709615574949

**Published:** 2015-02-18

**Authors:** Laura Travan, Maria Santa Rocca, Francesca Buonomo, Lisa Cleva, Vanna Pecile, Angela De Cunto

**Affiliations:** 1 Institute for Maternal and Child Health, IRCCS “Burlo Garofolo”, Trieste, Italy

**Keywords:** trisomy 9q, SNP array analysis, duplication, NTRK2

## Abstract

Chromosomal abnormalities may cause growth failure before or since birth. 9q duplication is reported as a cause of intrauterine growth restriction, mild dysmporphism, and intellectual disabilities. We report a case of a maternally inherited 9q21.31q21.33 duplication causing prenatal and postnatal growth restriction with feeding refusal and mild facial dysmorphisms, prenatally diagnosed by single-nucleotide polymorphism array analysis. Hypothesis of the possible pathogenic mechanisms are discussed.

## Short Clinical Summary and Investigations

We report a case of a maternal inherited 7.5 Mb duplication on chromosome 9q21.31q22.33, causing early growth restriction with feeding refusal and mild facial dysmorphisms, prenatally detected by single-nucleotide polymorphism (SNP) array analysis.

The patient was the second offspring of nonconsanguineous parents. Her sibling was apparently normal; her 34-year-old mother presented with intrauterine growth restriction (birth weight 2950 g, gestational age 42 weeks) and similar feeding difficulties when she was a child. Moreover, she suffered from language delay and auto-aggressive behavior at school age. The mother also had one previous spontaneous abortion. Physical examination of the mother showed mild dysmorphic features.

The patient was born at 40 weeks gestational age through spontaneous vaginal delivery. Birth weight was 2785 g (3rd to 10th percentile), length 48 cm (10th to 25th percentile), and cranial circumference 34 cm (25th to 50th percentile). Prenatal history was uneventful until 20 weeks gestational age, when symmetric intrauterine growth restriction with disproportion between femur length (75th percentile) and cerebellar transverse diameter (45th percentile) was recognized; fetal head circumference and abdomen circumference were below the 5th percentile. A 3-dimensional ultrasound scan during third trimester suggested fine fetal facial dysmorphisms. Fetal karyotype was normal and Silver–Russell syndrome was not verified by uniparental disomy (UDP) analysis.

**Figure 1.**
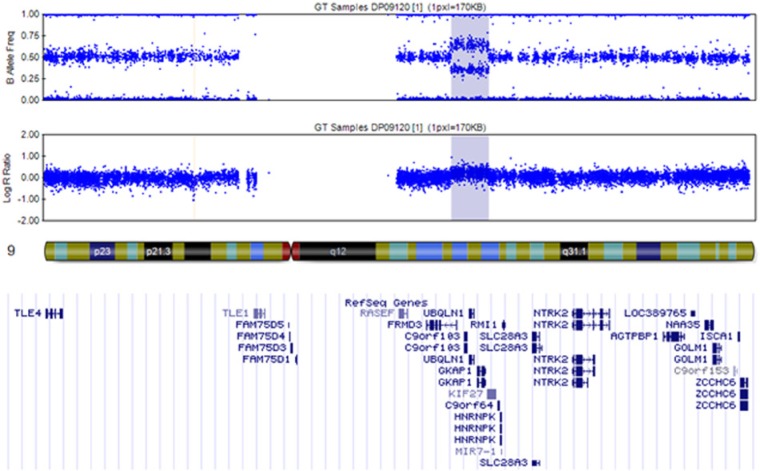
Single nucleotide polymorphism (SNP) arrays revealing a 7.5 Mb duplication of the chromosome 9q21.31q22.33.

Molecular karyotype analysis performed on cultured amniocytes by high-resolution SNP arrays revealed a 7.5 Mb duplication of the chromosome 9q21.31-q21.33. The duplication included approximately 28 genes (Figure 1), among which NTRK2 (neurotrophic tyrosine kinase, receptor, type 2) gene (genomic coordinates of the duplication: 81,693,987 to 89,271,532 (GRCh37)). Molecular karyotyping of the mother revealed the same duplication of the chromosome 9(q). Sibling karyotype was normal. No other living relatives were available for molecular analysis. The patient was followed up until 2-year-old showing a persistent feeding refusal and failure to thrive evident since the first months of life, in spite of the use of hypercaloric formulas. Other causes of failure to thrive—including celiac disease—were excluded.

**Figure 2.**
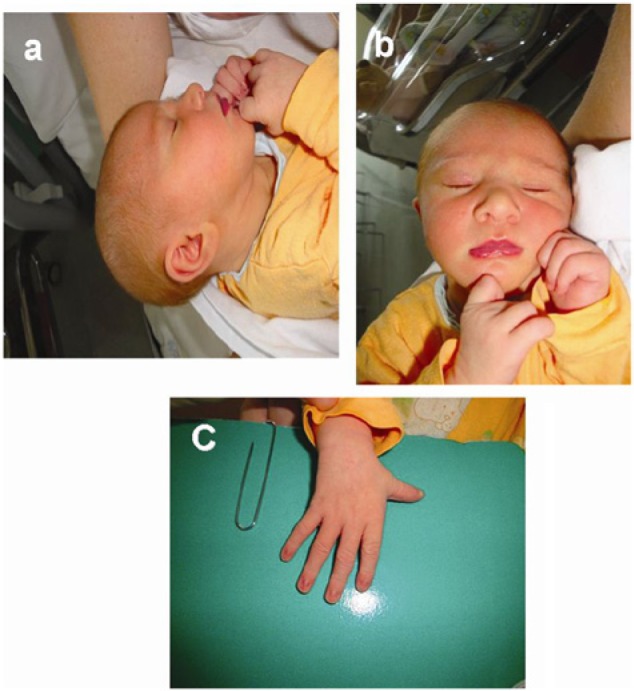
Dysmorphic features showed by the proband: (panel A) low set and large ears; (panel B) hypertelorism, bulbous nasal tip with short nasal bridge, and thin upper lip; and (panel C) proximal placement of thumb and long and thin fingers.

Auxological assessment showed always a weight under the 3rd percentile with a length on the 75th percentile; physical examination confirmed mild dysmorphic features similar to those presented by the mother: hypertelorism, bulbous nasal tip with short nasal bridge, low-set and large ears, long and thin fingers, thin upper lip (Figure 2), proximal placement of thumb, hypoplastic and deep-set nails and nipples abnormalities. Developmental milestones were almost normal except for a mild language delay.

## Discussion

Failure to thrive in early life can be due to numerous causes, including malabsorption, increased metabolic demand, and failure of the child to take sufficient calories. Chromosomal abnormalities usually cause symmetric growth failure before or since birth. Interestingly, our patient presented feeding refusal with low weight gain but normal length.

Partial trisomy of 9q has been associated with a constellation of symptoms, including short stature, intellectual disability, microcephaly, facial dysmorphisms, pyloric stenosis, and various defects of the heart, distal extremities, eye, thyroid, and esophagus.^1-4^

To our knowledge, to date 8 cases of 9q duplication involving a similar genomic region and detected by SNP arrays are reported in databases DECIPHER and ECARUCA. Among the 5 patients reported in DECIPHER, phenotypic features are described only in 3 cases: Intellectual disabilities were reported in 2 out of 3 (259328, 261794), whereas the third case (256837) presents a wider duplication and therefore is not comparable to our child. Three cases are reported in the ECARUCA database. Although all of them present wider 9q duplication (genomic coordinates: q21.13-qter; q21.2-22.1; q21.2-22.3), they share some phenotypic characteristics with our patient. Similar dysmorphic features are reported in all of them (thin upper lip, nipple abnormalities, proximal placement of thumb, hypoplastic and deep-set nails, bulbous nasal tip, low-set ears), intrauterine growth restriction was present in 2 of 3 patients and 1 patient suffered from speech delay.

Chromosome 9 shows great architectural variation with several regions containing tandem repeats which predispose to deletion and duplication. However, the pathological features presented by our patient are similar to those reported in other cases of 9q duplication and the size of genomic region duplicated was large (7.5 Mb), supporting the hypothesis that the duplication found is not a normal variant.

NTRK2 gene is located on 9q21.33, involved in our patient’s duplication. NTRK2 encodes a member of the neurotrophic tyrosine receptor kinase family (TrkB). This kinase is a membrane-bound receptor that, on neurotrophin binding (brain-derived neurotrophic factor [BDNF]), phosphorylates itself and members of the MAPK (mitogen-activated protein kinase) pathway. TrkB receptors are expressed in hypothalamic nuclei and seem to be involved in the regulation of mammalian eating behavior and energy balance.^5,6^ Moreover, a heterozygous de novo mutation in the NTRK2 causing a loss of function has previously been shown in a patient with early-onset obesity, hyperphagia, and severe developmental delay.^7,8^ Activation of BDNF/TrkB signaling reduces food intake and body weight gain as BDNF acts as a catabolic agent. Although it was not possible to investigate the gene product and caution is needed in interpreting the results of genetic analysis, the hypothesis that duplication of NTRK2 may have a key role in feeding refusal in our case seems to be fascinating and may have potential clinical and scientific implications.

Interestingly, we detected the same mutation in the patient’s mother. Very few cases of partial trisomy 9 have been reported in young adults^9,10^ and to our knowledge this is the first case diagnosed prenatally. TrkB has been genetically proven to play an important role in hippocampal synaptic plasticity and learning^11,12^ and has been proposed as a susceptibility factor for autism,^13^ suggesting that *NTRK2* duplication in the patient’s mother may have influenced both feeding difficulties and behavioral phenotype.

In conclusion, the duplication in 9q21.31q21.33 could be a cause of feeding refusal and early growth restriction, and should be considered if specific mild dysmorphisms are present in infants with failure to thrive. Moreover, aberrant expression of a single gene involved in feeding behavior and food intake among a wide duplication represents a fascinating new model to explain genetic failure to thrive. SNP arrays could represent a useful tool both prenatally (in case of intrauterine growth restriction) and in early life (in case of early feeding refusal) as chromosomal analysis are less likely to give an answer in the absence of major malformations or dysmorphisms.

Written informed consent was obtained from the parents for publication of this case report.
